# Supplementary figures and images for: Approaches to induce the maturation process of human induced pluripotent stem cell derived-endothelial cells to generate a robust model
Source: PLoS One. 2024 Feb 23;19(2):e0297465. doi: 10.1371/journal.pone.0297465 (PMC10889888; doi:10.1371/journal.pone.0297465)

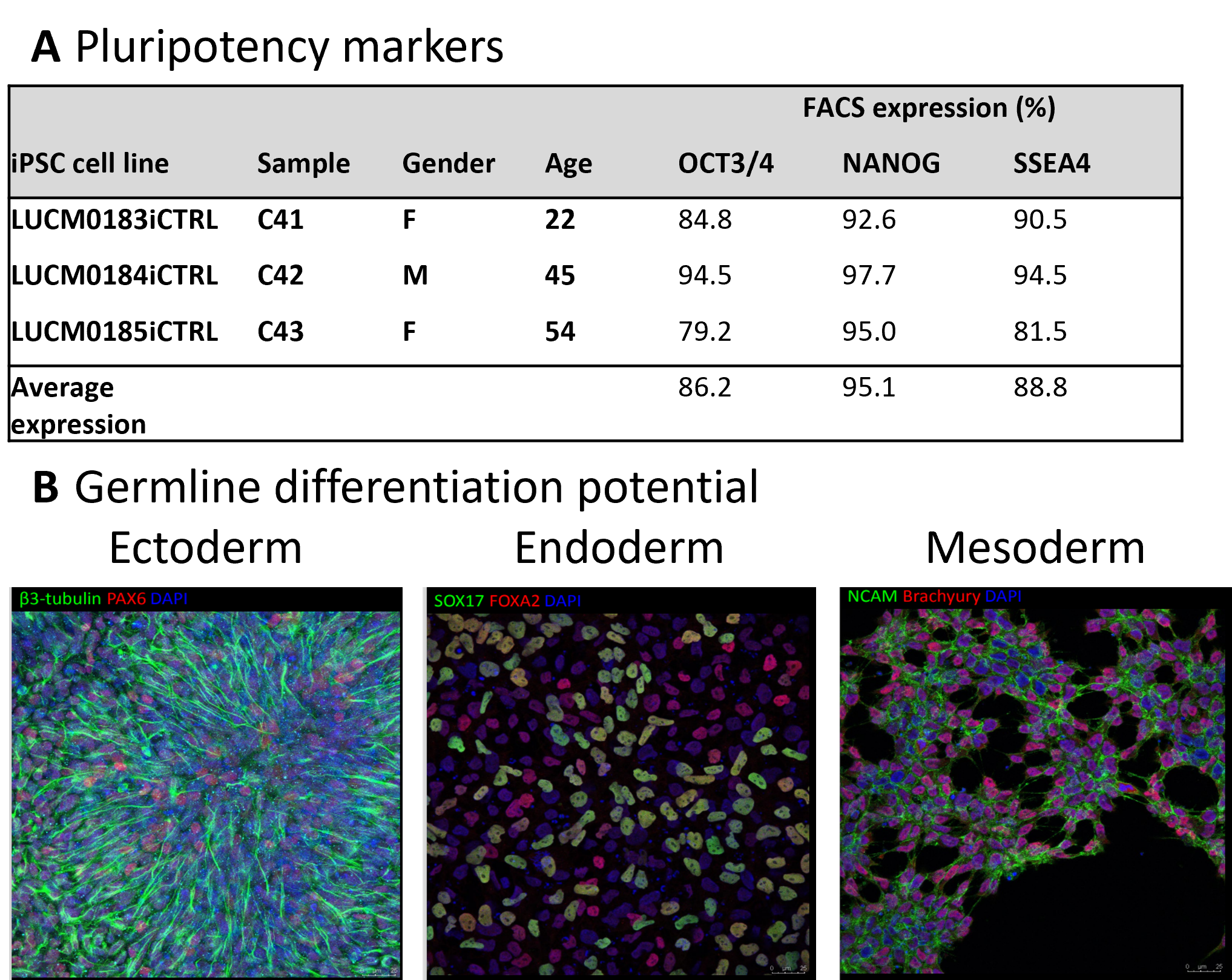

Supplement: S1 Fig — A) Donor information and FACS data of pluripotency markers. B) IF images of markers showing differentiation potential into the three germlines. (TIF) [file pone.0297465.s001.tif]

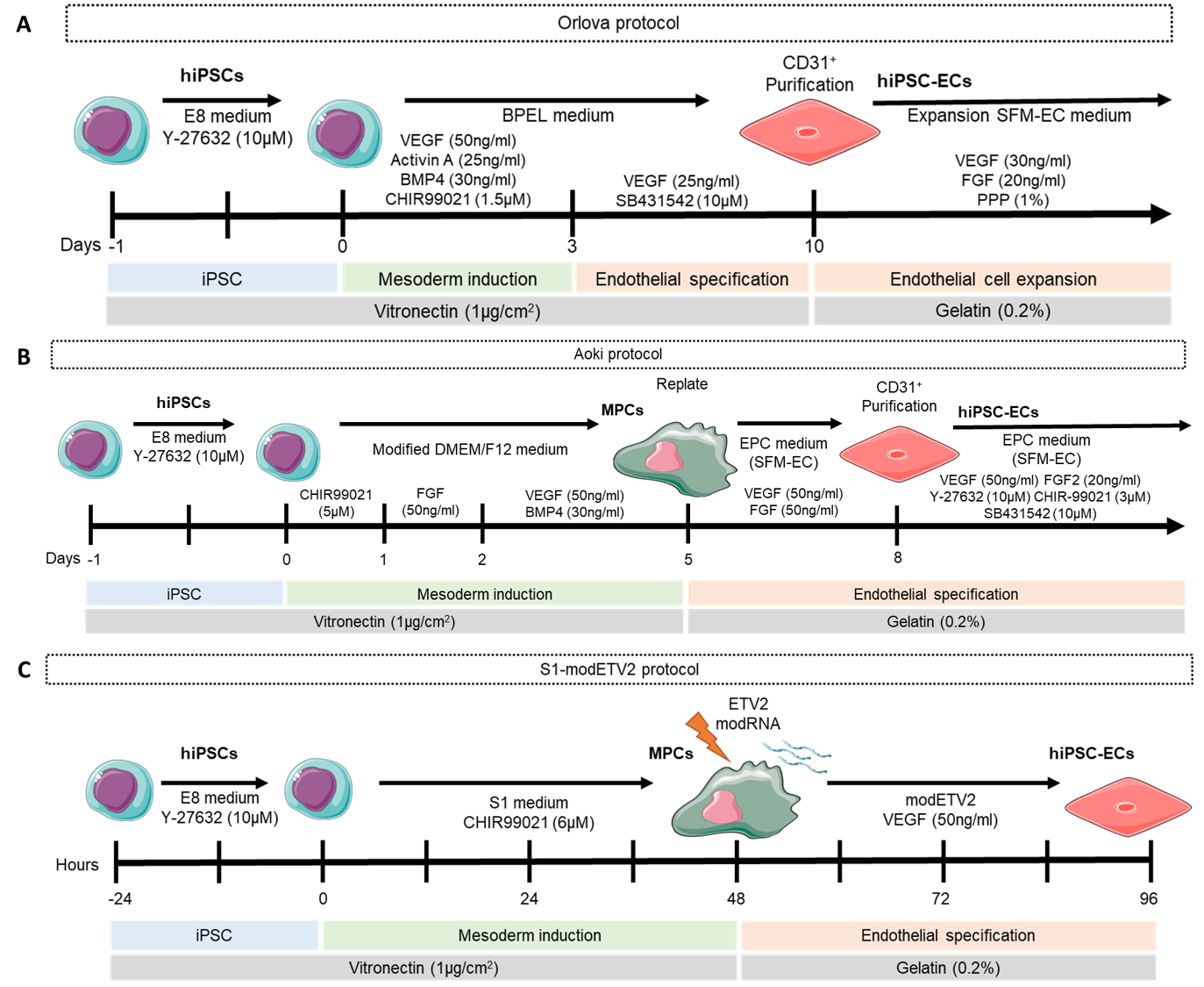

Supplement: S2 Fig — A) Orlova protocol. B) Aoki protocol. C) ETV2 protocol. (TIF) [file pone.0297465.s002.tif]

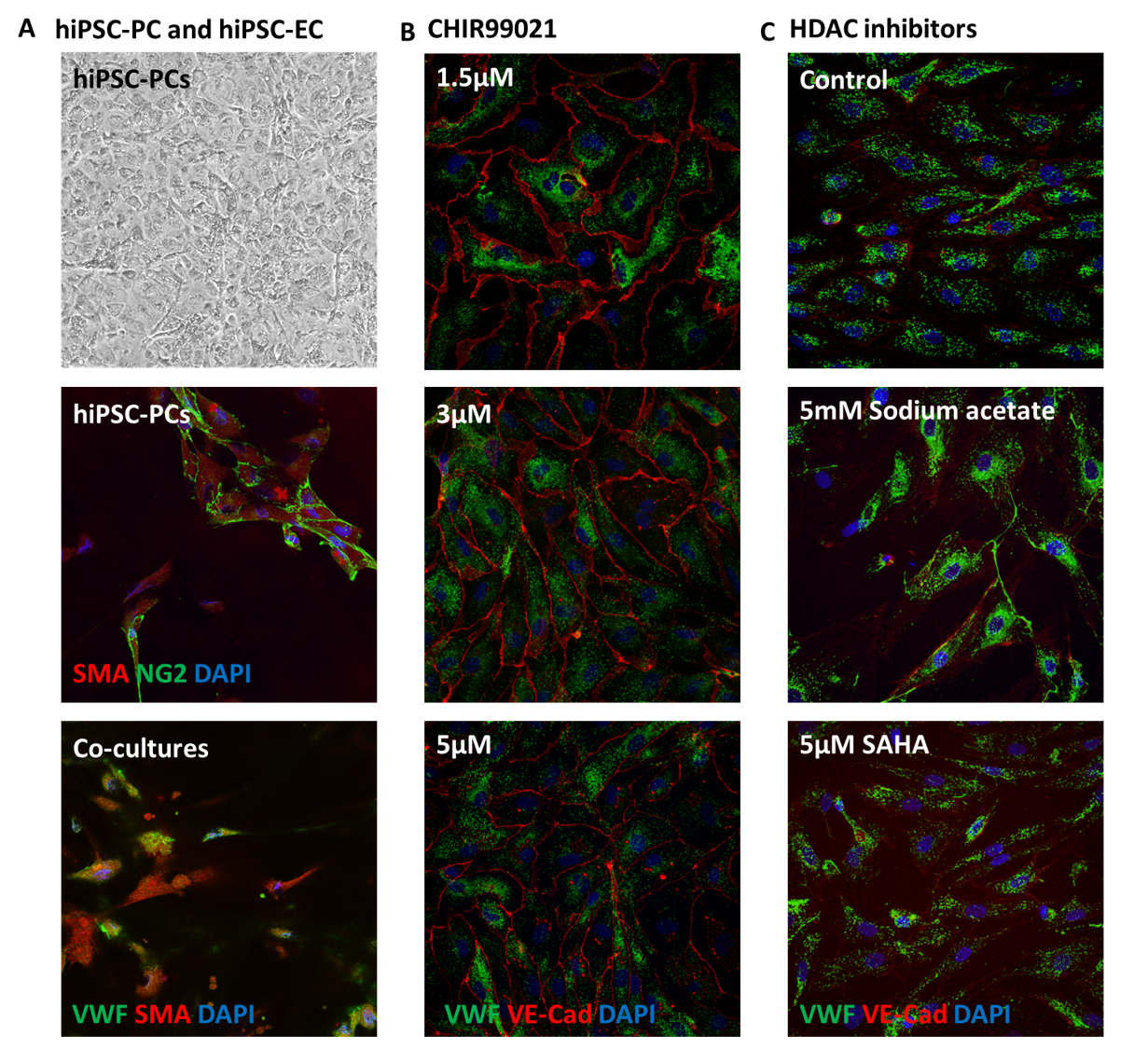

Supplement: S3 Fig — A) hiPSC-EC cocultures with hiPSC-PCs. B) Varying concentration of endothelial differentiation factor CHIR99021. C) Addition of different HDAC inhibitors. (TIF) [file pone.0297465.s003.tif]

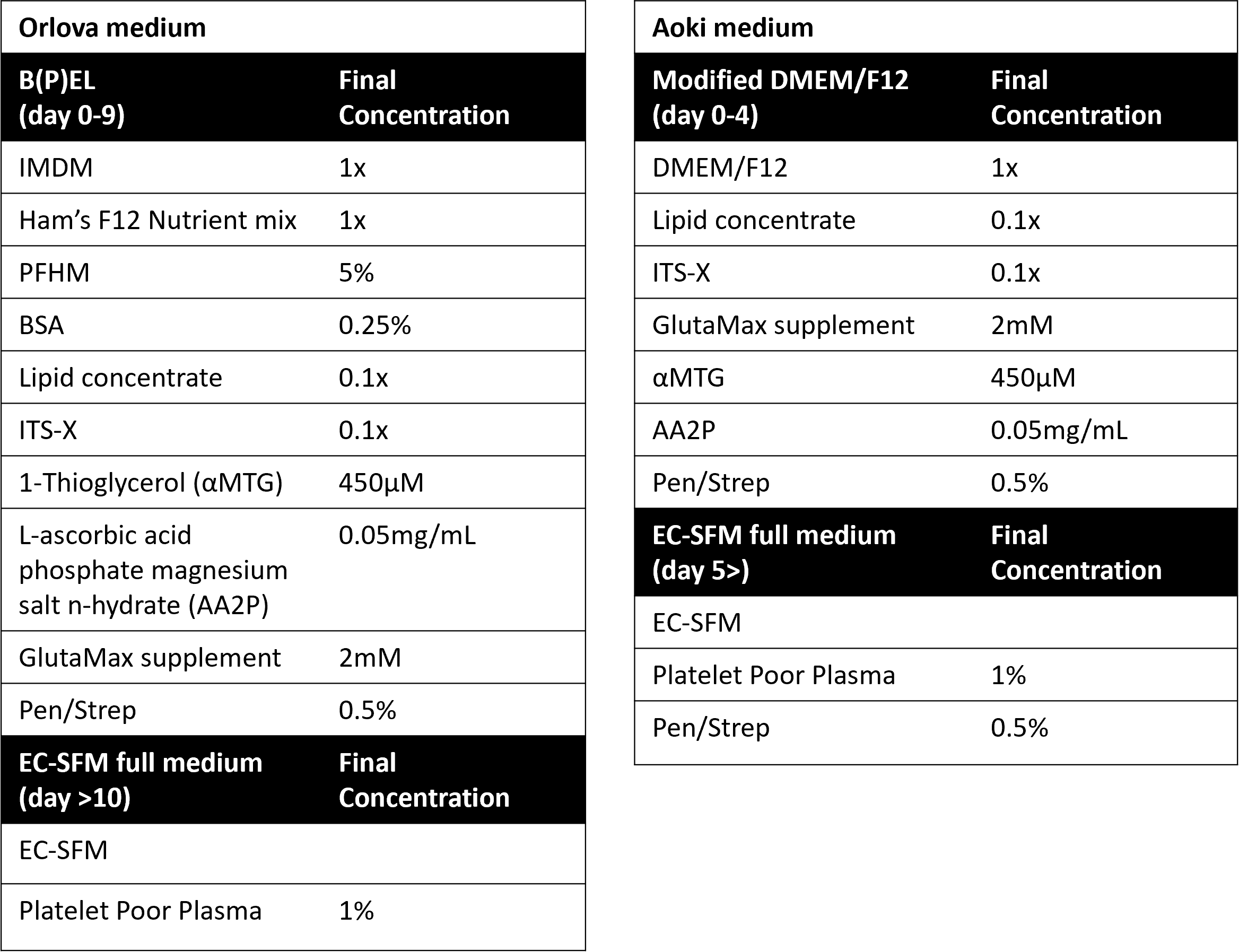

Supplement: S1 Table — (TIF) [file pone.0297465.s004.tif]
